# Digestive autoimmune diseases mimicking gastrointestinal manifestations in children with sickle cell anemia: A report of three cases

**DOI:** 10.1002/jpr3.70062

**Published:** 2025-07-13

**Authors:** Saray Mesonero Cavia, Marta García Bernal, Maria Jose López Liñan, Roger García Puig

**Affiliations:** ^1^ Department of Pediatric Gastroenterology, Hepatology, and Nutrition Mutua Terrassa University Hospital Terrassa Barcelona Spain; ^2^ Department of Pediatric Hematology and Oncology Mutua Terrassa University Hospital Terrassa Barcelona Spain; ^3^ Department of Pediatric Gastroenterology, Hepatology, and Nutrition Consorci Sanitari de Terrassa Hospital Terrassa Barcelona Spain

**Keywords:** autoimmune hepatitis, comorbidity in pediatrics, diagnostic challenges, hemoglobin S disease, inflammatory bowel disease

## Abstract

Sickle cell anemia (SCA) is a genetic disorder that presents with a variety of systemic complications, including gastrointestinal (GI) manifestations. These GI symptoms can overlap with those of digestive autoimmune diseases (DAD) such as inflammatory bowel disease (IBD) and autoimmune hepatitis (AIH), complicating the diagnosis and management. This study presents three cases of SCA patients diagnosed with DAD, highlighting the diagnostic challenges and therapeutic outcomes. Our goal is to stress how abdominal symptoms in SCA can mask the presence of DAD, leading to potential misdiagnoses. We review the implications of these findings in clinical practice and compare them to the literature to emphasize the importance of recognizing and differentiating these conditions to avoid delays in treatment.

## Abstract (highlights)

**What is Known:**

- Sickle cell anemia (SCA) often presents with gastrointestinal symptoms that complicate diagnosis.
- Digestive autoimmune diseases are rare but significant complications in pediatric SCA.
- Differentiating abdominal symptoms related to SCA from autoimmune gastrointestinal diseases can be challenging.

**What is New:**

- Significant delays in diagnosing co‐occurring digestive autoimmune diseases were observed in three pediatric SCA cases. This was attributed to symptom overlap that complicated differentiation from medication side effects or vaso‐occlusive crises.
- Emphasizes the importance of recognizing autoimmune hepatitis and inflammatory bowel disease in pediatric patients with SCA.
- Provides insights to enhance differential diagnosis and reduce therapeutic delays in SCA cases with persistent gastrointestinal symptoms.

## 1 INTRODUCTION

Sickle cell anemia (SCA) is a genetic disorder caused by mutations in the hemoglobin gene, leading to the production of hemoglobin S. This results in the sickling of red blood cells, causing a variety of systemic complications, including in the pulmonary, neurological, renal, cardiovascular, and gastrointestinal (GI) systems. Among these, vaso‐occlusive crises (VOC) are a major cause of morbidity. VOC can lead to recurrent episodes of ischemia and reperfusion injury, often affecting the GI tract and presenting as abdominal pain, bloody stools, and other nonspecific GI symptoms.

A less recognized complication in children with SCA is the development of digestive autoimmune diseases (DAD), such as inflammatory bowel disease (IBD) and autoimmune hepatitis (AIH). This overlap can create diagnostic confusion, potentially delaying appropriate treatment. For instance, *Lynch* et al. emphasized the higher prevalence of AIH among SCA patients compared to the general population and discussed pathophysiological links, such as the inflammatory role of free heme and immune dysregulation.^1^ Waisbourd‐Zinman et al. reported on the association between SCA and autoimmune liver disease (AILD), including AIH and sclerosing cholangitis (SC), highlighting the difficulty in distinguishing these conditions from common SCA‐related hepatopathies like hepatic sequestration and intrahepatic cholestasis.^2^ *Tamire and Million* documented the rare coexistence of ulcerative colitis (UC) and SCA in an Ethiopian child, emphasizing the importance of comprehensive diagnostic evaluation in patients presenting with recurrent abdominal pain and chronic diarrhea.^3^ Adepoju et al. documented the coexistence of SCA and IBD, including cases where initial misdiagnosis delayed effective treatment.^4^ Similarly, *Alqoaer* et al. reported chronic colitis mimicking IBD in an 11‐year‐old with SCA, demonstrating the need to differentiate ischemic colitis from inflammatory pathology in this population.^5^ This study aims to explore the coexistence of SCA with DAD, specifically focusing on IBD and AIH,^6^ and to discuss the diagnostic challenges in distinguishing these conditions from GI issues related to SCA, such as ischemic colitis caused by VOC.

## 2 METHODS

We reviewed case reports up to December 2023 of SCA patients diagnosed with DAD from two hospitals near Barcelona (Consorci Sanitari de Terrassa Hospital and Mutua de Terrassa Universitary Hospital). The cases included diagnoses of IBD and AIH, with diagnosis based on endoscopic and histological findings for IBD. UC is characterized by continuous inflammation limited to the colon, with symptoms like bloody diarrhea and abdominal cramping. Diagnosis is confirmed through colonoscopy, revealing mucosal inflammation, and histology, showing neutrophils and crypt abscesses.^7^, ^8^ Crohn's disease (CD) involves discontinuous, transmural inflammation throughout the GI tract, with symptoms like abdominal pain, diarrhea, and weight loss. Diagnosis requires colonoscopy, identifying deep ulcers and granulomas, and imaging to assess small bowel involvement.^9^ Both conditions are confirmed by clinical, endoscopic, histological, and radiological evaluations, with extra‐intestinal manifestations supporting diagnosis. AIH diagnosis follows the ESPGHAN criteria, including elevated liver enzymes, IgG, and the presence of interface hepatitis on liver biopsy.^10^ Data analysis focused on clinical presentation, diagnostic challenges, and treatment responses.

Additionally, a literature review was conducted to explore the relationship between these autoimmune entities and SCA, focusing on pathophysiological mechanisms, clinical presentations, and diagnostic challenges.^1^, ^2^, ^3^, ^4^, ^5^, ^6^, ^11^

### 2.1 Ethics statement

This short communication was conducted in accordance with the ethical standards of the Declaration of Helsinki. The study is based on anonymized clinical cases for which written informed consent was obtained from the patients’ legal guardians. The collection and use of data comply with current regulations on data protection and ethical research practices. The “CEIM Fundació Assistencial Mútua Terrassa” has established that coded data may be used for research purposes without compromising patient identity. All identifying information has been removed or anonymized to ensure patient confidentiality. The authors adhered to institutional guidelines and protocols in the collection, analysis, and reporting of the data.

## 3 RESULTS

### 3.1 Case 1

**Figure 1:**
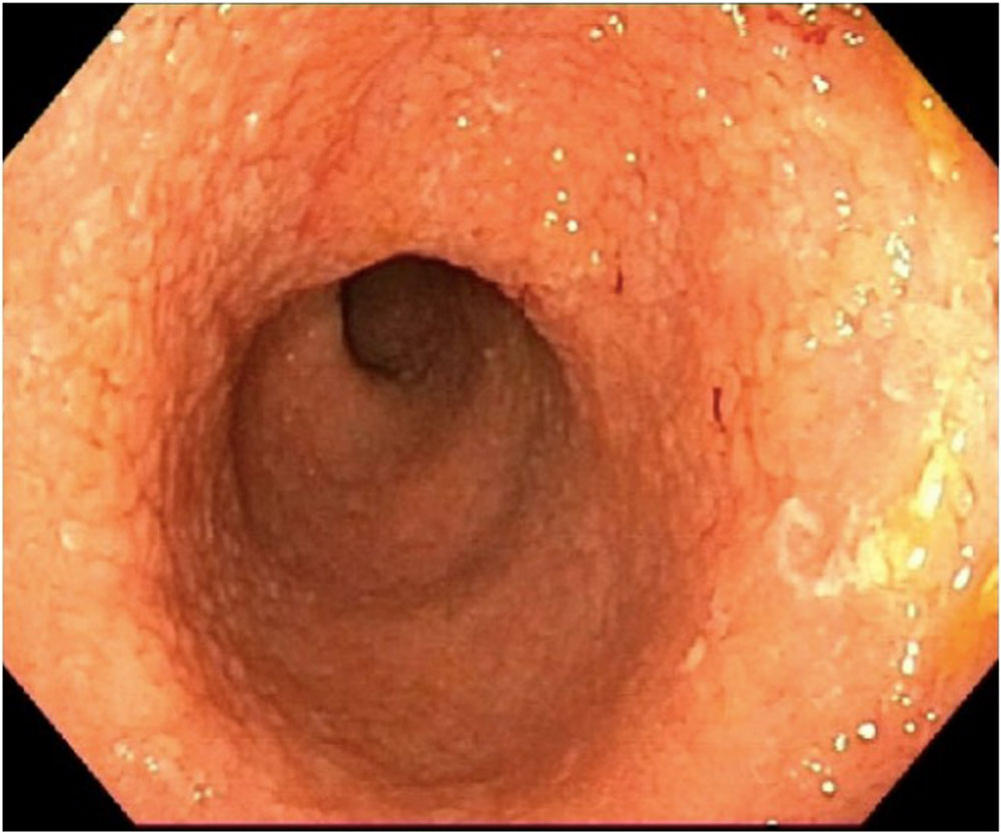
Colonoscopic findings of ulcerative colitis showing mucous erythema, vascularity loss and a regenerative pattern.

An 8‐year‐old Senegalese boy with SCA (HbSS) presented with chronic diarrhea lasting over 2 years and weight stagnation. During follow‐up with the hematology and gastroenterology services, serial blood tests were performed. The patient experienced multiple VOC with significant episodes of anemia, leading to the initiation of treatment with hydroxyurea. In some blood tests, a mild intermittent elevation of transaminases and gamma‐glutamyl transferase was observed. It was ruled out that hydroxyurea was the cause of their gastrointestinal symptoms. He had no abdominal pain, fever, tenesmus, or vomiting. Initially, fecal calprotectin was normal, but in 6 months it rose to 2600 µg/g) and occult blood was positive. Esophagogastroduodenoscopy (EGD) was normal, while colonoscopy revealed pancolitis with erythematous mucosa, loss of vascularity, and small ulcers (Figure 1). Biopsy confirmed chronic active colitis without crypt abscesses. Despite initial improvement with 5‐ASA and azathioprine, the patient failed to respond to anti‐TNF therapy (Infliximab and then Adalimumab), as well as accelerated Ustekinumab regimen, and is now on vedolizumab, with a good response. An enterography and cholangiopancreatography (MRCP) were performed to rule out other possible gastrointestinal complications, given the complex presentation and to evaluate for potential ischemic changes or biliary issues, though both were normal.

### 3.2 Case 2

**Figure 2:**
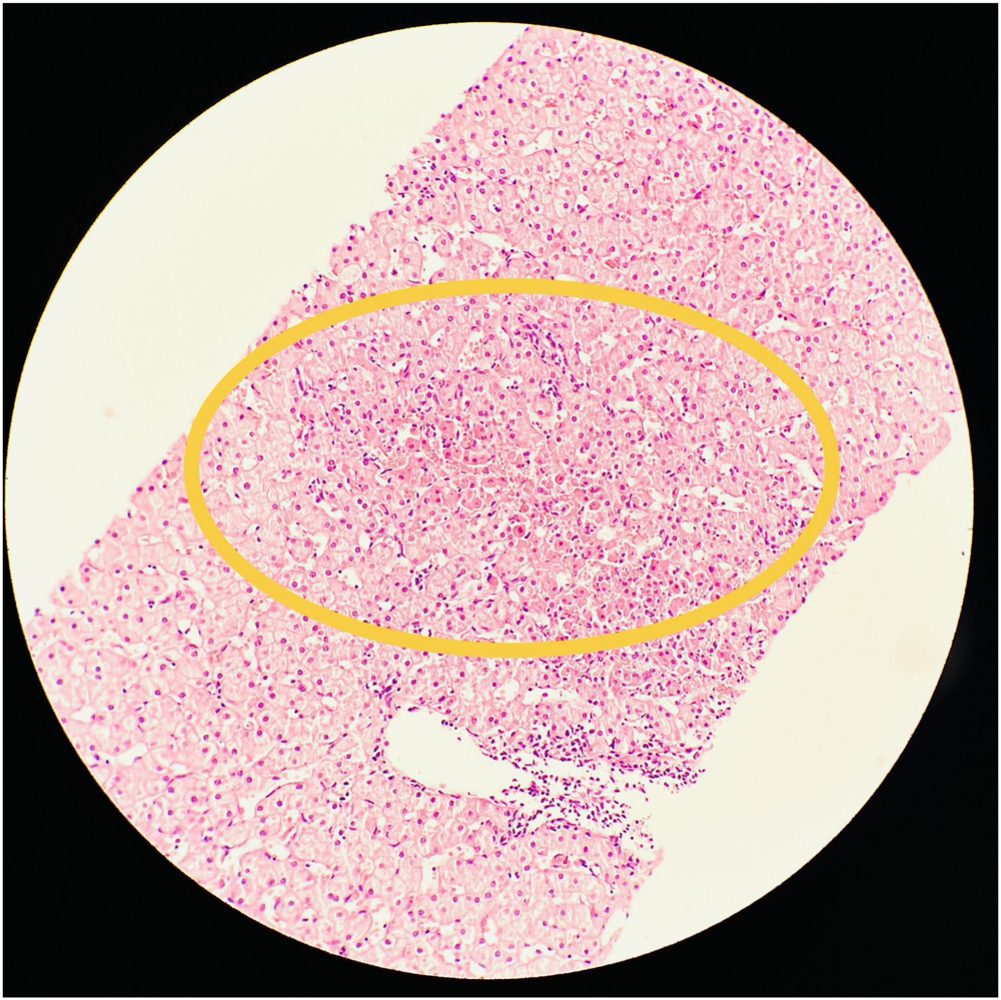
Histologic findings of the liver biopsy showing mild lymphocytic infiltration and lobular inflammatory activity with focal hepatocyte necrosis.

A 9‐year‐old Senegalese girl with SCA (HbSS) had a history of recurrent abdominal pain and poor weight gain over 4 years. She frequently visited the emergency department for pain, which resolved within hours. Liver enzyme tests revealed persistent transaminase elevation, along with hyperbilirubinemia and increased gamma‐glutamyl transferase. Abdominal ultrasound and viral serologies were normal, and celiac disease was ruled out. MRCP was normal. Positive anti‐smooth muscle antibodies led to a liver biopsy, which confirmed AIH (Figure 2). The patient was treated with oral steroids, followed by azathioprine, and underwent a compatible human leukocyte antigens (HLA) bone marrow transplant (BMT), which cured both AIH and SCA. The patient has maintained normal liver enzymes and blood counts without further treatment.

### 3.3 Case 3

A 15‐year‐old Moroccan girl with SCA (HbSS) experienced multiple admissions for hemolytic and vaso‐occlusive crises. She had persistent abdominal pain and constipation for 4 years. Choledocholithiasis was diagnosed and treated with MRCP, laparoscopic cholecystectomy, and ursodeoxycholic acid. Despite this, her abdominal pain persisted, with negative infectious stool studies. Physical examination revealed a linear intergluteal fissure and hypertrophic anal papilla. Fecal calprotectin intermittently peaked at 1800 µg/g. Endoscopic studies, including capsule endoscopy and colonoscopy, revealed two small duodenal ulcers and non‐necrotizing epithelioid granulomas in the perianal area, confirming CD. The patient was treated with oral budesonide followed by azathioprine, achieving complete remission. She is currently on 100 mg of azathioprine daily and remains asymptomatic.

## 4 DISCUSSION

This report underscores the challenges in diagnosing DAD, such as IBD and AIH, in patients with SCA, where abdominal symptoms may be attributed to VOC or ischemic colitis. As highlighted in *Lynch* et al.'*s* systematic review, liver dysfunction in SCA is often misattributed to hemolysis or iron overload, potentially delaying the diagnosis of autoimmune conditions such as AIH.^1^ *Waisbourd‐Zinman* et al. highlighted the diagnostic complexity of AILD in SCA, stressing that elevated liver enzymes in SCA patients should prompt evaluation for autoimmune liver disease beyond the common causes such as choledocholithiasis or VOC.^2^ The three cases presented illustrate the complex interplay between SCA and DAD, highlighting the importance of considering both conditions in the differential diagnosis when SCA patients present with GI symptoms. The potential for misdiagnosis is significant, as GI symptoms in SCA can mimic or overlap with those of autoimmune diseases, leading to delays in appropriate treatment.^4^

The cases presented here align with findings in the literature. For example, in Case 1, initial management as UC failed due to the overlap in clinical features with ischemic colitis, a phenomenon also described by *Tamire and Million*.^3^ The eventual response to vedolizumab underscores the importance of tailored therapeutic approaches in these patients. Case 2 presented the potential role of bone marrow transplantation (BMT) as a curative strategy for AIH and SCA.

As in other autoimmune diseases, the role of stem cell transplantation in the management of IBD has been described, both with autologous and allogeneic sources.^1^, ^2^, ^6^, ^12^ There are numerous references in the scientific literature on the role of allogeneic hematopoietic stem cell transplantation in cases of monogenic inflammatory bowel disease in younger children, as well as in Crohn's disease refractory to multiple treatments.^13^, ^14^, ^15^ However, the use of hematopoietic stem cell transplantation in patients with ulcerative colitis is poorly described in the literature.^16^

On the other hand, in cases of coexistence of a haematopathy tributary of HSCT and IBD in the same patient, the presence of IBD does not lead to a worse outcome, neither to higher rates of implant failure or graft‐versus‐host disease.^17^, ^18^

A promising stem cell treatment modality in these patients consists of the use of mesenchymal cells derived from bone marrow stem cells, both in Crohn's Disease and Ulcerative Colitis.^19^

In Case 3, CD was diagnosed after persistent GI symptoms, and elevated fecal calprotectin prompted further investigation, consistent with the diagnostic approach described by *Alqoaer* et al.^5^ *Adepoju* et al. also highlighted the importance of thorough diagnostic evaluations to differentiate IBD from ischemic or infectious colitis in SCA patients.^4^

The therapeutic overlap between managing SCA and DAD also poses challenges. The response to immunosuppressive treatments in our cases mirrors outcomes reported in the literature, where corticosteroids and disease‐modifying agents effectively induce remission in colitis associated with SCA.^3^, ^4^, ^11^ However, the potential role of curative approaches like BMT remains an area requiring further investigation.

## 5 CONCLUSION

The limited number of published cases of SCA and autoimmune diseases highlights the potential underdiagnosis of these conditions. Increased awareness of these potential associations is critical to prevent misdiagnosis and ensure that patients receive the most appropriate care. Recognizing the possibility of DADs in SCA patients with recurrent GI symptoms will help clinicians avoid overlooking ischemic events like colitis ischemica and autoimmune conditions, potentially reducing morbidity and improving patient outcomes.

## CONFLICT OF INTEREST STATEMENT

The authors of this short communication, based on three case reports, certify that there is no conflict of interest, financial or otherwise, related to the content of this manuscript. No financial support or relationships with commercial entities have influenced the study design, data collection, interpretation, or writing of this report. The findings presented are the result of independent academic research, with no external biases or conflicts that could have affected the outcomes or conclusions.

## Data Availability

The data supporting the findings of this communication are available from the corresponding author upon reasonable request. Due to patient privacy and confidentiality concerns, some data may be restricted or de‐identified in compliance with ethical standards. Access to the full data set will be considered for legitimate research purposes, subject to the necessary institutional approvals and patient consent.

## References

[1] Lynch K , Mega A , Piccin A , Daves M , Fogarty H . Liver disease & sickle cell disease: autoimmune hepatitis more than a coincidence; a systematic literature review. Mediterr J Hematol Infect Dis. 2023;15(1):e2023060. 10.4084/MJHID.2023.060 38028400 PMC10631714

[2] Waisbourd‐Zinman O , Frenklak R , Hakakian O , Hilmara D , Lin H . Autoimmune liver disease in patients with sickle cell disease. J Pediatr Hematol Oncol. 2021;43(7):254‐257. 10.1097/MPH.0000000000001985 33181587

[3] Tamire AH , Million T . Sickle cell disease with ulcerative colitis in an Ethiopian child. Int Med Case Rep J. 2024;17:521‐525. 10.2147/IMCRJ.S453861 38799385 PMC11127645

[4] Adepoju AA , Akere A , Ogun GO , et al. Co‐existing sickle cell anaemia and inflammatory bowel disease: case report and review of the literature. Paediatr Int Child Health. 2022;42(1):29‐35. 10.1080/20469047.2021.1936393 34474658

[5] Alqoaer K , Ahmed MM , Alhowaiti ES . Inflammatory bowel disease in a child with sickle cell anemia. Case Rep Pediatr. 2014;2014:732785. 10.1155/2014/732785 25093137 PMC4100385

[6] Chuang E , Ruchelli E , Mulberg AE . Autoimmune liver disease and sickle cell anemia in children: a report of three cases. J Pediatr Hematol Oncol. 1997;19(2):159‐162. 9149749 10.1097/00043426-199703000-00013

[7] Turner D , Ruemmele FM , Orlanski‐Meyer E , et al. Management of paediatric ulcerative colitis, part 1: ambulatory care—an evidence‐based guideline from European Crohn's and Colitis Organization and European Society of Paediatric Gastroenterology, Hepatology and Nutrition. J Pediatr Gastroenterol Nutr. 2018;67(2):257‐291. 10.1097/MPG.0000000000002035 30044357

[8] Turner D , Ruemmele FM , Orlanski‐Meyer E , et al. Management of paediatric ulcerative colitis, part 2: acute severe colitis—an evidence‐based consensus guideline from the European Crohn's and Colitis Organization and the European Society of Paediatric Gastroenterology, Hepatology and Nutrition. J Pediatr Gastroenterol Nutr. 2018;67(2):292‐310. 10.1097/MPG.0000000000002036 30044358

[9] Van Rheenen PF , Aloi M , Assa A , et al. The medical management of paediatric Crohn's disease: an ECCO‐ESPGHAN guideline update. J Crohns Colitis. 2021;15(2):171‐194. 10.1093/ecco-jcc/jjaa161 33026087

[10] Mieli‐Vergani G , Vergani D , Baumann U , et al. Diagnosis and management of pediatric autoimmune liver disease: ESPGHAN Hepatology Committee position statement. J Pediatr Gastroenterol Nutr. 2018;66(2):345‐360. 10.1097/MPG.0000000000001801 29356770

[11] Hasosh T , Eliakim R , Koren I , et al. A case of crohn's disease associated with sickle cell anemia: challenges in diagnosis and management. Inflamm Bowel Dis. 2016;22(9):E34‐E37.27508516

[12] Nagaishi K , Arimura Y , Fujimiya M . Stem cell therapy for inflammatory bowel disease. J Gastroenterol. 2015;50(3):280‐286. 10.1007/s00535-015-1040-9 25618180

[13] Morita M , Takeuchi I , Kato M , et al. Intestinal outcome of bone marrow transplantation for monogenic inflammatory bowel disease. Pediatr Int. 2022;64(1):e14750. 10.1111/ped.14750 33884705

[14] Tomomasa D , Suzuki T , Takeuchi I , et al. Successful allogeneic hematopoietic cell transplantation for patients with IL10RA deficiency in Japan. J Clin Immunol. 2024;Sep 12 45(1):6. 10.1007/s10875-024-01795-6 39264505

[15] McDonald GB , Landsverk OJB , McGovern DPB , et al. Allogeneic bone marrow transplantation for patients with treatment‐refractory Crohn's Disease. Heliyon. 2024;10(1):e24026. 10.1016/j.heliyon.2024.e24026 38283244 PMC10818189

[16] Unnikrishnan A , Glover SC , Norkina O , Wingard JR , Norkin M . Complete resolution of severe ulcerative colitis after haploidentical hematopoietic stem cell transplantation followed by post‐transplant high‐dose cyclophosphamide. Bone Marrow Transplant. 2017;52(8):1204‐1205. 10.1038/bmt.2017.94 28581471

[17] Rabian F , Porcher R , Sicre de Fontbrune F , et al. Influence of previous inflam matory bowel disease on the outcome of allogeneic hematopoietic stem cell transplantation: a matched‐pair analysis. Biol Blood Marrow Transplant. 2016;22(9):1721‐1724. 10.1016/j.bbmt.2016.05.022 27246370

[18] Peric Z , Peczynski C , Polge E , et al. Influence of pretransplant inflammatory bowel disease on the outcome of allogeneic hematopoietic stem cell transplantation: a matched‐pair analysis study from the Transplant Complications Working Party (TCWP) of the EBMT. Bone Marrow Transplant. 2021;56(12):3084‐3087. 10.1038/s41409-021-01458-9 34561559

[19] Lightner AL , Dadgar N , Matyas C , et al. A phase IB/IIA study of remestemcel‐L, an allogeneic bone marrow‐derived mesenchymal stem cell product, for the treatment of medically refractory ulcerative colitis: an interim analysis. Colorectal Dis. 2022;24(11):1358‐1370. 10.1111/codi.16239 35767384 PMC9795998

